# Impact of COVID-19 pandemic on dental education: online experience and practice expectations among dental students at the University of Jordan

**DOI:** 10.1186/s12909-021-02584-0

**Published:** 2021-03-08

**Authors:** Susan Hattar, Abeer AlHadidi, Faleh A. Sawair, Islam Abd Alraheam, Ahmad El-Ma’aita, Fouad Kadim Wahab

**Affiliations:** 1 Department of Conservative Dentistry, School of Dentistry, The University of Jordan, Amman, 11942 Jordan; 2 Department of Oral Maxillofacial Surgery, Oral Medicine and Periodontology, School of Dentistry, The University of Jordan, Amman, Jordan

**Keywords:** COVID-19, Dental education, Online learning, Preparedness

## Abstract

**Background:**

The quarantine associated with the COVID-19 pandemic forced dental schools to suspend their clinical training and to shift to distant learning methods. The aim of this study is to investigate the impact of quarantine on the self-perceived preparedness of dental graduates and to explore the efficacy of online education from students’ perspectives.

**Methods:**

The questionnaire distributed to dental students comprised of two main sections. The first part covered the online education experience, and the second part measured the level of self-perceived preparedness for a range of cognitive, communication and professional skills.

**Results:**

The survey yielded a response rate of 72%. The majority of students (77%) agreed that they missed educational experiences as a result of the lockdown. More than half of them felt less motivated to follow-up with distant e-learning and believed that online assessment is not a good method for evaluation. A high percentage of the students (66%) thought that online group discussions had a positive value while 67% preferred online lectures compared to theatre lectures. Majority of students particularly 5th year (78.7%) (*p* < 0.001) stated that the quarantine increased their collaboration with their colleagues. According to 87% of students, the experience most negatively affected was their clinical training. In general, students showed satisfactory self-perceived preparedness related to a range of attributes and professional skills.

**Conclusions:**

The data showed that students partially appreciated the online system, whereas they did not consider it a substitute for face to face clinical practice. The overall self-perceived preparedness level was promising; however students had reservations regarding independent practice following graduation.

## Background

The gravity of Covid-19 pandemic has led governments and institutions to take draconian measures with regard to the ongoing education. In an attempt to balance the safeguarding of students, faculty, and patients while keeping track of the changing national policies, universities were forced to take different measures to ensure the continuity of education. One of the biggest challenges has been to stop direct patient care, which is a key component of the dental curriculum. One cannot deny that didactic and clinical skills are two different outcomes of education. No virtual sessions can duplicate the close experience with patients [1]. In all situations, it remains the responsibility of dental schools to certify the competency of dental students while being flexible to deviate from the original curricular plan. Dental associations around the world have developed the frameworks for dental education to ensure the finest academic practice for undergraduate education [2]. These councils define dentists as being scientifically oriented, professionally minded practitioners [3], who adhere to high standards of ethical and professional conduct and who can practice safely as a members of the healthcare system [4]. These criteria represent our baseline outcomes that cannot be compromised.

During the past few months, e-learning had become the sole and only option to ensure the continuity of higher education. Fortunately, e-learning has proven to be a successful adjunct and has definitely impacted the environment in which medical students learn [5–9]. A preliminary study aimed at investigating the immediate response of European Dental Institutions showed that the vast majority of schools have reported using online pedagogical methods for non-clinical teaching, while permitting very limited clinical activities [10]. This study demonstrated the need for evidence-based information to help decision-makers and educators reform and construct the new face of dental education. Our institution, as many others, started to embed e-learning and blended learning into specific courses. Some disciplines seem to function well this way; however, this cannot be applied in its pure form for all subspecialties. For instance, in disciplines where patient care is a primary goal, students need to attend clinics and provide patient treatment while acquiring their competencies. Although new technologies with simulation and virtual reality techniques are gaining more interest, the direct in person experience is still one that cannot be simply replaced.

From another perspective, distant learning has multiple facades that can undoubtedly improve education [9, 11]. When compared to traditional didactic methods, the scientific material can be easily updated and rapidly accessed. Another advantage is that it can easily foster self-learning skills. Indeed, some studies have indicated that self-regulated learning significantly affects academic achievement and learning performance [12]. For e-learning to be effective, staff development, assessment strategies and technological elements must be well planned and standardized. Yet, the sudden shift from traditional methods of teaching to a more creative distant learning did not allow sufficient time for adaptation. In our institution, clinical sessions have been suspended and we ended up finishing the scholastic year with a substantial degree of cut down on student clinical exposure by approximately 30% of their final years of training. A question arises as to whether the fulfilment of learning outcomes for general practice was established through the online experiences. Our students have missed out on many valuable opportunities, such as case presentations, OSCE exams, yearly faculty conferences, in addition to their clinical sessions. Furthermore, addressing the psychological impact of this pandemic on students’ mental wellbeing is another issue that should not be overlooked. Isolated in quarantine, students are undoubtedly suffering from anxiety at different levels [1, 13, 14]. The fear of the unknown, the social distancing and the direct impact of COVID 19 on their future practice. We ask ourselves in what way students will perceive themselves in the real practical environment to evolve and integrate in their careers.

From another view, the general preparedness of dental students is influenced by many factors, such as curriculum design; training model; teaching methods and the overall educational environment. The keystone skills and attributes indispensable for graduation have been documented. Nevertheless, we acknowledge that the current lack of adequate undergraduate training might reflect on the overall preparedness and confidence of our graduating students.

Resilience is the keyholder for success in these challenging times. To leverage technological advances to our benefit, implement new technologies that could raise the bar of education. Staff should also follow up with students to further help them consolidate their knowledge and facilitate their self-directed learning with innovative strategies. These approaches will help us circumvent the havocs to our benefit.

We believe that it is opportune time to reflect upon the effect of the pandemic on dental education and training. From this perspective, a survey was developed to explore the outcome of this shortened and modified scholastic year on the students’ acquired skills and future aspirations. The first objective was to investigate the efficacy of distant learning from students’ perspective and to pinpoint the attributes that were mostly affected whether clinical, cognitive or behavioral skills. The second objective was to measure the student self-perceived preparedness in relation to cognition, communication, and professional skills. Furthermore, a preview of current challenges associated with distant education and possible futuristic reforms were discussed.

## Methods

### Data collection

The study protocol was reviewed and approved by the Institutional Review Board of Jordan University Hospital (Ref # 75/2020/616).

At the end of scholastic year 2019–2020, all fifth-year graduates (*N* = 193) and fourth year (*N* = 239) dental students were asked to complete a questionnaire developed using (Qualtrics®) before their final examinations. To maintain the anonymity, no personal identifiers were used in the online questionnaire. The introduction of the questionnaire defined the purpose and objectives of the study. The authors also stated clearly that participation is completely voluntary with no penalties associated with refusal or withdrawal from participation. Consent was implied by responding to the questionnaire.

The questionnaire (Appendix) comprised of two main themes/sections. The first section which was distributed to both fifth and fourth-year students was designed to investigate the students’ attitudes regarding online learning. Questions were related to their educational experience with e-learning, namely the efficacy of this system, their engagement level, their collaboration with colleagues, the value of this system in comparison with traditional methods and finally the assessment methods applied. Finally, data regarding the experience and the subspecialty that had the highest negative impact were gathered. The responses of this section were reported on a four-point Likert scale (strongly agree, agree, disagree, strongly disagree).

The second section investigated students’ self-perceived preparedness for dental practice. This part was distributed only to graduating students of the 2019–2020 batch (*N* = 193). It had two main categories. The first category consisted of 17 questions derived from a previously validated scale [15] that explores a broad range of skills and attributes expected from dental students at the time of graduation. These 17 indicators of cognition, communication, and professional skills had 3 scales: “No experience” which was allocated a score of zero; “Mostly” which was allocated a score of one, and “Always” which was allocated a score of two. The total score for the self-perceived preparedness scale ranged from 0 to 34.

The second category focused on students’ perceived readiness for dental practice with regards to confidence in skills acquired before graduation, preference to be monitored after graduation, readiness for independent practice and predilection for a well-structured year of residency.

### Statistical analysis

Statistical analysis was performed using SPSS for Windows release 16.0 (SPSS Inc., Chicago, IL, USA). Descriptive statistics were generated, and Chi-square test and independent sample t-test were used to examine differences between groups. The significance level was stated as *P* < 0.05.

## Results

A total of 310 participants responded to the online questionnaire, yielding a response rate of 72%. The study sample was composed of 179 (57.7%) fourth year and 131 (42.3%) fifth year dental students. They were composed of 241 (77.7%) female and 69 (22.3%) male students.

### Section 1: impact on dental education. Students’ attitudes towards online learning

The educational impact of COVID-19 on 4th and 5th year dental students is shown in Table 1.

**Table 1:** Educational impact of COVID-19 on 4th and 5th year dental students

Question		Year	Strongly disagree %	Disagree %	Agree %	Strongly agree %	*P* value*
1	Do you feel that you missed educational experiences as a result of the lockdown?	4th	3.4	20.7	49.7	26.3	0.96
		5th	3.8	18.3	51.1	26.7	
		female	4.1	19.9	51.5	24.5	0.392
		male	1.4	18.8	46.4	33.3	
		Total	3.5	19.7	50.3	26.5	
2	Do you think online assessment is a good method for evaluation?	4th	22.9	43.0	30.2	3.9	**0.001**
		5th	13.0	30.5	50.4	6.1	
		female	19.9	40.2	35.3	4.6	0.106
		male	14.5	29.0	50.7	5.8	
		Total	18.7	37.7	38.7	4.8	
3	Do you think group discussion posted on E-learning such as clinical cases and scenarios had a positive value on your education?	4th	14.0	34.6	44.7	6.7	**< 0.001**
		5th	0.8	14.5	51.1	33.6	
		female	9.5	27.4	44.4	18.7	0.196
		male	4.3	21.7	58.0	15.9	
		Total	8.4	26.1	47.4	18.1	
4	Did this quarantine increase your collaboration with your colleagues?	4th	6.1	41.9	33.5	18.4	**< 0.001**
		5th	1.5	19.8	55.0	23.7	
		female	3.7	33.6	41.9	20.7	0.797
		male	5.8	29.0	44.9	20.3	
		Total	4.2	32.6	42.6	20.6	
5	Did you feel more engaged and motivated in following up with distant e-learning?	4th	19.6	39.1	30.2	11.2	**0.049**
		5th	14.5	33.6	45.0	6.9	
		female	16.6	36.5	36.1	10.8	0.415
		male	20.3	37.7	37.7	4.3	
		Total	17.4	36.8	36.5	9.4	
6	Do you prefer online lectures compared to face to face theatre lectures?	4th	10.1	27.4	38.5	24.0	0.14
		5th	4.6	22.1	48.1	25.2	
		female	6.2	24.5	44.0	25.3	0.241
		male	13.0	27.5	37.7	21.7	
		Total	7.7	25.2	42.6	24.5	
7	Do you feel comfortable with all this technology-based education?	4th	13.4	40.2	35.8	10.6	**< 0.001**
		5th	1.5	33.6	55.7	9.2	
		female	8.3	40.2	43.6	7.9	0.064
		male	8.7	27.5	46.4	17.4	
		Total	8.4	37.4	44.2	10.0	

*p value of Chi square test

The majority (76.8%) of the students, without significant effects of gender or year of study, agreed that they missed educational experiences as a result of the lockdown.

More than half (56.4%) of the students, particularly 4th year (65.9%) (*p* = 0.001), feel that online assessment is not a good method for evaluation. Similarly, 54.2% of the students, particularly 4th year (58.7%) (*p* = 0.049), feel less engaged and motivated in following-up with distant e-learning.

In contrast, a higher percentage of the students (65.5%), particularly the 5th year (84.7%) (*p* < 0.001), think that group discussion posted on e-learning such as clinical case scenarios had a positive value on their education. Similarly, a higher percentage of the students (63.2%), particularly the 5th year (78.7%) (*p* < 0.001), think that the quarantine increased their collaboration with their colleagues. In addition, 67.1% of the students, without significant effects of gender or year of study, prefer online lectures compared to face to face theatre lectures. A significantly higher percentage of 5th year dental students (64.9%) compared to 4th year dental students (46.4%) (*p* < 0.001) felt comfortable with the technology-based education.

**Fig. 1:**
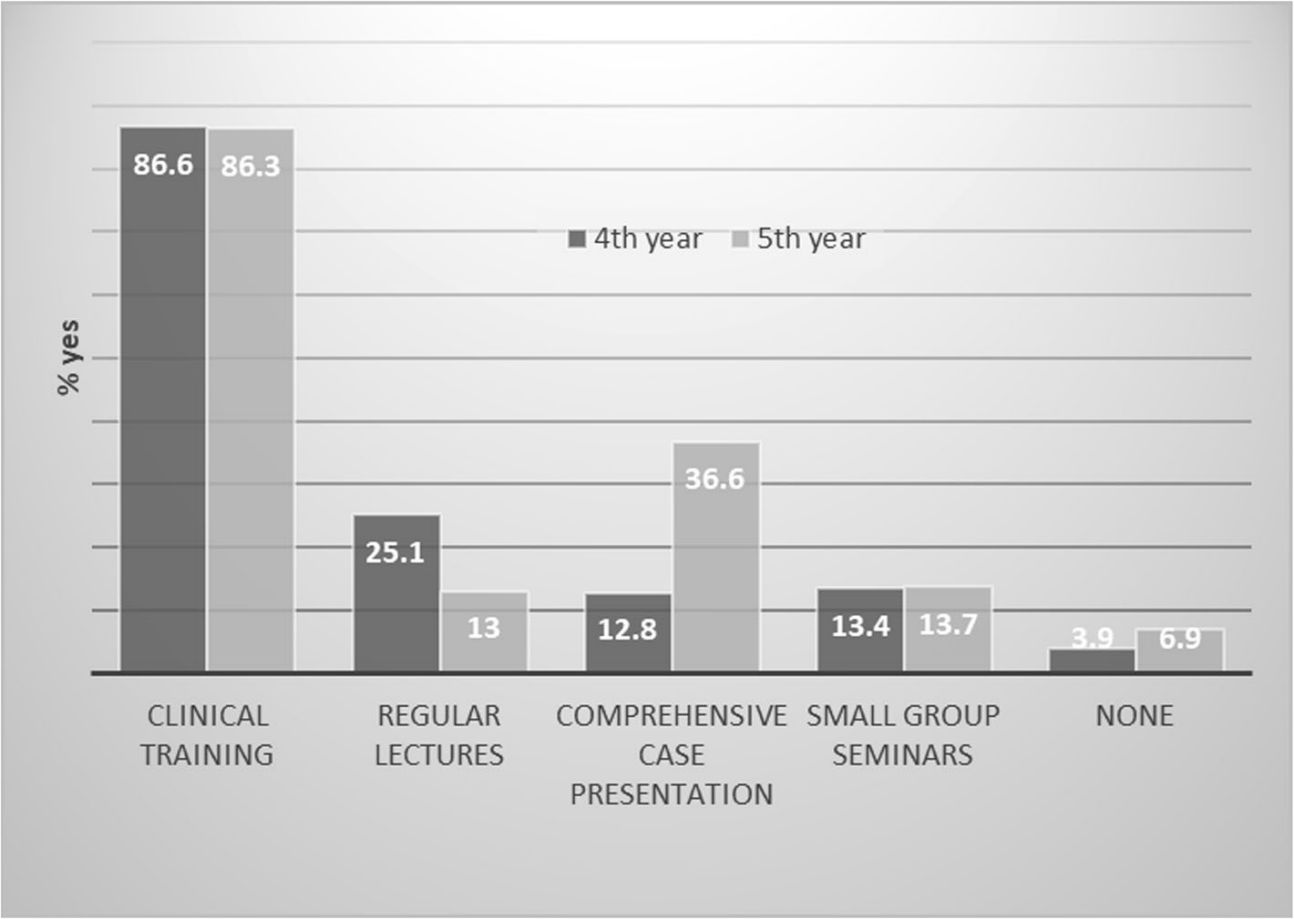
Experiences mostly affected by quarantine

The experiences that were mostly affected by quarantine according to 4th and 5th year dental students are shown in Fig. 1. Clinical training was the most affected experience reported by students (86.5%). Regular lectures were missed by a significantly higher percentage of 4th year students (*p* = 0.008), while comprehensive case presentations were missed by a higher percentage of 5th year students (*p* < 0.001).

**Fig. 2:**
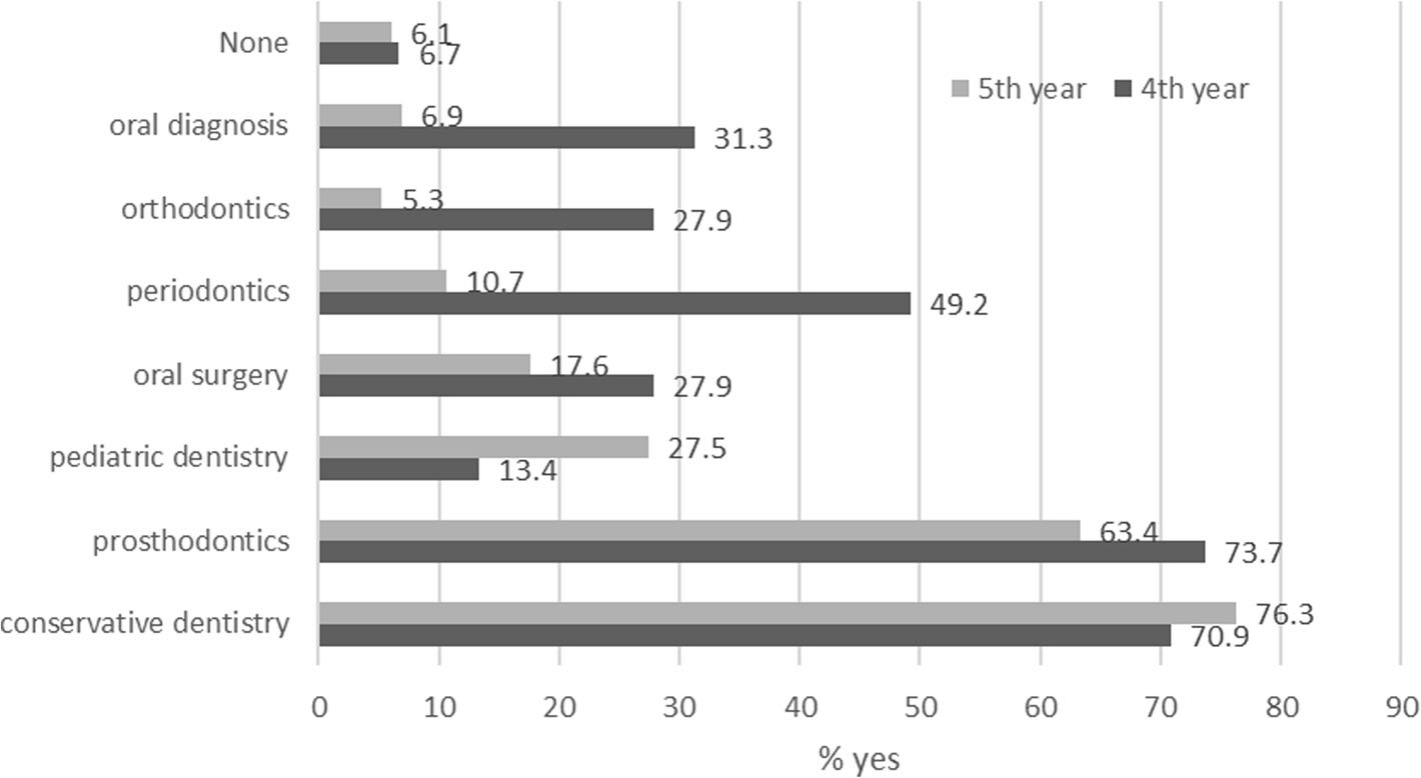
Dental subspecialties most negatively affected by quarantine

According to 4th and 5th year dental students, the dental subspecialties most negatively affected by quarantine are shown in Fig. 2. Conservative dentistry (73.2%) followed by prosthodontics (69.4%) were the two most negatively affected subspecialties while orthodontics (18.4%) and oral diagnosis (21.0%) were the two least negatively affected subspecialties. Pediatric dentistry (*p* = 0.002) was reported to be negatively affected by higher percentage of 5th year students, while oral surgery (*p* = 0.033), periodontics (*p* < 0.001), orthodontics (*p* < 0.001), and oral diagnosis (*p* < 0.001) were reported to be negatively affected by higher percentage of 4th year students.

### Section 2: self-perceived preparedness related to cognition, communication, and professional skills

The mean total self-perceived preparedness score (TPS) for the 131 participants 5th year students was 24.4 (SD ±4.7) with a range of 5 to 34. The mean TPS was not significantly affected by the gender of the participants (*p* > 0.05).

The responses of the students to the 17 indicators of self-perceived preparedness related to cognition, communication, and professional skills are shown in Table 2. Generally, students perceived a total or most of time preparedness related to the majority of attributes and professional skills. However, a significant percentage of students find difficulty in evaluating new dental materials using an evidence-based approach (47.3%) and in having sufficient evidence-based knowledge of scientific principles to support their dental practice. In addition, approximately 10% of students do not reflect on their clinical practice in order to address their learning needs.

**Table 2:** Self-perceived preparedness related to cognition, communication and professional skills of 5th year dental students arranged in descending order with regards to “No experience”

Skill		No experience %	Mostly %	Always %
1	I evaluate new dental materials/products using evidence-based approach.	47.3	38.9	13.7
2	I have sufficient evidence-based knowledge of scientific principles to support my practice.	19.1	66.4	14.5
3	I reflect on my clinical practice in order to address my learning needs.	9.9	50.4	39.7
4	I can manage patient’s expectations for their treatment.	7.6	61.1	31.3
5	I am able to refer patients with complex treatment needs.	6.9	30.5	62.6
6	I feel comfortable asking for help from supervisor/colleague if needed.	6.9	46.6	46.6
7	I maintain accurate records of my clinical notes.	4.6	50.4	45
8	I restrict my relations with my patients to a professional level.	4.6	43.5	51.9
9	I take responsibility for my continuing professional development.	3.8	33.6	62.6
10	I take appropriate measures to protect patient confidentiality.	3.8	22.9	73.3
11	I can communicate potential procedural risks to my patients.	3.1	47.3	49.6
12	I can obtain informed consent from my patients.	2.3	31.3	66.4
13	I can motivate my patients to maintain good oral/general health.	1.5	55	43.5
14	I am aware of my legal responsibilities as a dental professional.	0.8	31.3	67.9
15	I recognize my personal limitations in clinical practice.	0.8	39.7	59.5
16	I can communicate appropriately with my colleagues.	0.8	26.7	72.5
17	I can communicate effectively with my patients.	0	35.9	64.1

### General practice readiness

As shown in Table 3, nearly one-third of 5th year students are not sure of their confidence in the skills acquired before graduation and nearly two-thirds of them prefer to be mentored or indirectly supervised following graduation. In addition, only 28.2% have confidence in starting an independent practice after graduation and, therefore, the majority prefer to have a well-structured year of residence with proper training after graduation.

**Table 3:** Practice readiness of 5th year dental students

Question		No %	Unsure %	Yes %
1	Do you have confidence in the skills acquired before graduation?	3.8	35.1	61.1
2	Do you prefer to be mentored or indirectly supervised following graduation?	11.5	24.4	64.1
3	Do you have confidence in starting an independent practice after graduation?	27.5	44.3	28.2
4	Do you have preference for a well-structured year of residence, with proper training?	3.1	10.7	86.3

## Discussion

The shock that the pandemic caused to the entire educational system was abrupt and unprecedented. With very short notice, we found ourselves unable to teach conventionally, left with unorthodox resources and limited possibilities. Multidisciplinary approaches, clinical environments were abandoned, and teaching as we know it was suspended, with only one solution of being creative in an online environment. In many institutions, online teaching is still considered rudimentary while its clinical implementation is not evident, consequently teaching the medical clinical skills has become the ultimate challenge.

Little evidence has been reported regarding the plausible impact of this pandemic on higher education. One study has shown that there could be a positive effect of the COVID-19 confinement on students’ performance [16]. The authors concluded that the quarantine changed students’ learning strategies to a more efficient productive routine with higher levels of engagement. Contrary to their results, the majority of our students sensed that they actually missed important learning experiences especially the clinical sessions and felt less engaged in following up with distant learning. This could be explained by the fact that the former study was related to education in basic sciences, whereas dentistry needs practice and ample clinical exposure. One comparative survey administered to professional dental students indicated that students experienced increased level of stress and stated that the clinical education suffered from the pandemic [17]. This new teaching methodology does not come without barriers and is definitely not suitable for all courses. Furthermore, our participants stated that conservative dentistry was the subspecialty most negatively affected. This is comprehendible since the bulk of students’ clinical exposure during their final years revolves around restorative dentistry. Furthermore, a difference in students’ response regarding quarantine affected subspecialties was noted. For instance, 5th year students stated that oral surgery, periodontics, orthodontics and oral diagnosis were the least affected. This might be due to their ample clinical exposure in the previous academic year resulting in a higher sense of confidence and competence. For the purpose understanding the contributing factors to a confident graduation, one study conducting group interviews with students confirmed that the most important factor affecting their preparation was indeed their clinical exposure [18].

Growing evidence claims that e-learning is as effective as traditional methods [19]. A review investigating the barriers and enablers of e-learning concluded that distant education might enhance learning and performance due to its flexibility and accessibility [20]. Some studies have demonstrated higher satisfaction with e-learning methods and reported that many medical students find e-learning gratifying [11, 21]. Similar results from a study conducted in Italy demonstrated that e-Learning has been appreciated by students and professors in terms of teacher-student interactions, nonetheless clinical training cannot be totally replaced by remote activities [22]. Comparatively, our students seem to appreciate online lectures and forum case discussions which might be due to the interactive nature of these components. However, this is a self-perceived satisfaction that does not necessarily reflect higher performance [6, 9].

Although the teaching and learning arms of education might not have been dramatically affected, it is the assessment part that had the highest fluctuation in this new distant scenario. During our final exams, summative assessments of clinical reasoning are usually performed through Objective Structured Clinical Examinations (OSCEs). These exams have been widely used in medical field and substitute a valid methodology with high educational value [23, 24]. This year all on campus exams have been cancelled. From students’ perspective, the online substitute assessments did not match the traditional methods for evaluation. One causal factor could be the lack of proper tools to facilitate the process such as flawless connections at their locations, in addition to the anxiety built up from lack of direct presence of the evaluators.

The success of distant learning relies not only on the students’ motivation but also on continuous interaction between learners and facilitators [20]. A synchronous collaboration could be the cornerstone to enhance their level of engagement [25]. Our students have noticed that the quarantine increased their interactions and collaboration especially with their classmates. This was more significant for 5th year students possibly due to their higher level of social interaction, since they had a better chance to get acquainted with each other and to work together during the final years. It is noteworthy to say that students appreciated the group discussion posted on the platform, however this was noticed more among the 5th year students. This might be explained by the fact that these students had ample clinical exposure compared to 4th year students. They were also more familiar with case-based discussions and clinical scenarios. The open communication that we witnessed between students and staff could have built the mutual trust and facilitated their cooperation. As educators, our role lies within encouraging the students to stay connected and follow up on their education. Clear directions should be given, and students should not be left in the dark when it comes to decisions that will directly affect their education.

On a range of cognitive and behavioral attributes our students were asked about their readiness for general dental practice. Our results show that self-perceived preparedness for graduating students was satisfactory for a wide range of affective skills and traits. Our students showed confidence in communication and continuous development, similar to other studies [26–29] still, some skills regarding evidence-based practice and knowledge need consolidation and additional emphasis. This conforms with studies performed in different worldwide institutions where evidence-based dentistry and critical appraisal skills were lacking [15, 28, 30, 31]. We can still argue that the attitudes and knowledge of graduates may alter after embarking into the career world and that their concerns might be overcome once they integrate into their practice.

Many studies have shown that dental students are usually confident in carrying out procedures safely, nevertheless they felt somehow unprepared and still relied on some sort of supervision [29, 32]. Similarly, one third of our students were not sure of the skills acquired before graduation and nearly two-thirds preferred to be indirectly supervised following graduation. Mentoring was considered very important and they still had some reservations in regard to independent dental practice. This might be related to their doubts about the amount of practice they experienced during their final years. One way to overcome this anxiety would be to expose our students outreach teaching experiences such as community dental care, for it proved to be a useful adjunct for clinical experience, communication skills as well as self-confidence [33–35].

Navigating the challenges associated with this sudden shift, faculty members thrived to obtain the necessary training to use online platforms, yet not everyone has the same level of technological expertise. Not to mention, the necessary infrastructure and resources that are not always available to support this complete transition. Suddenly e-learning rose from an optional rudimentary choice to the only alternative at hand.

Dental institutions are now facing a defiant era with new guidelines implicated to ensure the safety of the dental team. Our responsibilities lie not only within providing adequate education but to make sure our students are competent to face the new work challenges. Clinical training cannot be substituted entirely by distant learning; therefore, the setting must be modified to allow a safe working place. With the lower clinical exposure our mission remains to ensure effective training. Personal protective measures must be applied to the highest standards. Patient triage and the use of SARS-CoV-2 diagnostic tests for staff, students and patients might prove beneficial for the sustainability of the workflow [22]. While the identification of the asymptomatic positive patient remains a challenge, also research validation of these serological tests is warranted [36].

Our findings may be considered as a beginning of a journey of investigations. Clear limitations such as the fact that this was a cross sectional survey performed at a single dental school cannot be neglected. Students in different schools with different online experience might respond differently. Additionally, the study was performed during an early phase when students suddenly found themselves in quarantine with a new context of education and new guidelines. Future wider perspective studies are deemed necessary. It would also be informative from an outcome point of view to compare educational results with those of previous years. We hope not to see this as a detriment to education but a learning experience giving way to a new generation of digital academia. Perhaps, this global change would create the momentum for us all to exchange ideas and share practices and valuable strategic initiatives [25]. New solutions such as simulation and virtual reality systems might be promising alternatives, while reemerging is the key to overcoming all challenges, for this pandemic allowed education to be reinvented.

## Conclusions

The global disruptions caused by the pandemic guided us to a new perspective which would be to bridge the gap of clinical training, and to find modified or alternative methods to ensure that students receive the necessary clinical experience. The pandemic implications did not only target education and patient care, but repercussions are witnessed in research, financial and psychological domains. Our role as academician is to ensure students’ preparedness, but it is equally important to understand work readiness from their perspective. Confinement was a new setting they have never experienced before, and the current online learning was a unique pedagogical experience. The data showed that students partially appreciated the new system with e-lectures and discussion forums, however they still believe that it was not a substitute for face to face clinical practice and they acknowledge missing many educational experiences. Their overall self-perceived preparedness level was promising, for majority of the attributes expected from dentists. However, their feedback reflected their reservation toward independent practice following graduation. This study emphasizes the importance of improving distant education and establishing collaborations between dental schools. Future research of new pedagogic methodologies is warranted.

## Data Availability

All data generated or analysed during this study are included in this published article.

## Appendix

### Section I: educational impact of online learning

1. Do you feel that you missed educational experiences as a result of the lockdown?
2. Do you think online assessment is a good method for evaluation?
3. Do you think group discussion posted on E-learning such as clinical cases and scenarios had a positive value on your education?
4. Did this quarantine increase your collaboration with your colleagues?
5. Did you feel more engaged and motivated in following up with distant e-learning?
6. Do you prefer online lectures compared to face to face theatre lectures?
7. Do you feel comfortable with all this technology-based education?
8. What is the experience that was mostly affected by your quarantine? you can choose multiple answers
  (Clinical training, Regular lectures, Comprehensive case presentation, small group seminars, none of the above)
9. Which subspecialty according to you was the most negatively affected? you can choose multiple answers
  (conservative dentistry, prosthodontics, pediatric dentistry, oral surgery, periodontics, orthodontics, oral diagnosis, none of the above)

### Section 2

#### Part 1: students self-perceived preparedness related to cognition, communication and professional skills

1. I evaluate new dental materials/products using evidence-based approach
2. I have sufficient evidence-based knowledge of scientific principles to support my practice
3. I reflect on my clinical practice in order to address my learning needs
4. I can manage patient’s expectations for their treatment
5. I am able to refer patients with complex treatment needs
6. I feel comfortable asking for help from supervisor/colleague if needed
7. I maintain accurate records of my clinical notes
8. I restrict my relations with my patients to a professional level
9. I take responsibility for my continuing professional development
10. I take appropriate measures to protect patient confidentiality
11. I can communicate potential procedural risks to my patients
12. I can obtain informed consent from my patients
13. I can motivate my patients to maintain good oral/general health
14. I am aware of my legal responsibilities as a dental professional
15. I recognize my personal limitations in clinical practice
16. I can communicate appropriately with my colleagues
17. I can communicate effectively with my patient

#### Part 2: practice readiness

1. Do you have confidence in the skills acquired before graduation?
2. Do you prefer to be mentored or indirectly supervised following graduation?
3. Do you have confidence in starting an independent practice after graduation?
4. Do you have preference for a well-structured year of residence, with proper training?
